# Evaluation of the potential virulence of *Mycobacterium avium* subspecies *paratuberculosis* isolates from cattle in Uganda

**DOI:** 10.1007/s42770-026-01951-7

**Published:** 2026-05-06

**Authors:** Judah Ssekitoleko, Franziska Tanneberger, Kamal H. Eltom, ElSagad Eltayeb, Uwe Truyen, Lonzy Ojok, Ahmad Amanzada, Edward Wampande, Moses Luutu Nsubuga, Peregrine Sebulime, Ahmed Abd El Wahed, Julius Boniface Okuni

**Affiliations:** 1 College of Veterinary Medicine, Animal Resources and Biosecurity, Makerere University, P. O. Box 7062, Kampala, Uganda; 2 Institute of Animal Hygiene and Veterinary Public Health, Leipzig University, Leipzig, D-04103 Germany; 3 Unit of Animal Health and Safety of Animal Products, Institute for Studies and Promotion of Animal Exports, University of Khartoum, Shambat, Khartoum North 13314 Sudan; 4 Faculty of Medicine, Al Neelain University/Ibn Sina Specialised Hospital Khartoum, Khartoum, 11112 Sudan; 5 Department of Livestock Health Research, Rwebitaba Zonal Agricultural Research and Development Institute, National Agricultural Research Organisation, P. O. Box 295, Entebbe, Uganda; 6 Department of Pathology, Faculty of Medicine, Gulu University, P.O. Box 166, Gulu, Uganda; 7 Department of Immunology and Molecular Biology, College of Health Sciences, Makerere University, P. O. Box 7062, Kampala, Uganda; 8 Department of Gastroenterology and Gastrointestinal Oncology, University Medical Centre Goettingen, Goettingen, D-37075 Germany

**Keywords:** Virulence, Cattle, Isolates, *Mycobacterium avium* subsp. *paratuberculosis*

## Abstract

Understanding the pathogenesis of paratuberculosis (PTB), caused by *Mycobacterium avium* subspecies *paratuberculosis* (MAP), is essential in the management of this economically important disease. MAP strains are known to differ genotypically, which could contribute to differences in strain virulence and disease outcomes. The behaviour of MAP inside the host and the mechanisms utilized by MAP to overcome the host defence system are not entirely understood. This study aimed at evaluating the potential virulence of different MAP isolates from cattle in Uganda. This was achieved by analysing the cytokine expression profiles of RAW 264.7 macrophages when infected with four different MAP isolates: MAP1, MAP2, MAP3 and MAP4. Specific real-time PCR was used to determine the expression of different cytokines, probable virulence genes of MAP as well as MAP quantities inside the macrophages at different time points (3 h, 24 h, 48 h) post-infection (pi). There were considerable variations in the cytokine expressions among macrophages infected with the different MAP isolates. The expression of IL-6 in macrophages infected with MAP1 at 48 h pi was noticeably high compared to the other isolates; and this is a known strategy for MAP survival inside macrophages. The regulation of the probable virulence genes: *kdpC*, *katG*, *papA2*, *impA*, *umaA1*, and the anti-apoptotic (*BCL-2*) gene also varied considerably among the isolates. Isolates MAP1, MAP2 and MAP3 demonstrated higher virulent behaviour regarding intracellular replication and survival compared to MAP4 isolate. The behaviour of these MAP isolates clearly indicates variations in virulence and further research needs to focus on how this can be exploited in the design of PTB diagnostic and control strategies.

**Supplementary Information:**

The online version contains supplementary material available at 10.1007/s42770-026-01951-7.

## Introduction

Paratuberculosis (Johne’s disease) caused by *Mycobacterium avium* subsp. *paratuberculosis* (MAP) is a serious and economically important disease in a wide range of animal species, especially ruminants. The infection leads to a chronic granulomatous enteritis which sometimes presents itself only after several years in form of severe wasting diarrhoea, resulting in cachexia and complete loss of productivity of the affected animal. Recently, the suspicion that MAP is a zoonotic pathogen has been strengthened [1, 2].

The disease is known to occur worldwide though the exact number of infections is only estimated and is in many cases probably higher than assumed. Early diagnosis of the disease is difficult due to the long period of time (often years), the animal takes before presenting clinical signs; yet it continues to spread the infection. The organism grows extremely slowly, and this complicates studies on its behaviour in host cells. Hence, prophylaxis for the disease is not normally successful due to the limited knowledge of the interaction of the organism with the host at the cellular level. Studies that targeted differentiation among MAP isolates distinguished two main genotypes or strains based on their growth characteristics and host associations: the “cattle type” or “Type C” and the “sheep type” or “Type S” [3]. A third type, the “Bison” or “B type”, was proposed, but was later designated as a subtype of Type C [4]. MAP has also been classified into type I, II and III based on whole genome sequence analysis of genetic markers [5]. These different strains and genotypes may contribute to variations in the clinical and pathologic presentation of the disease [6]. However, differential virulence in MAP strains has not yet been extensively investigated.

Microbial virulence is the ability of the pathogenic organisms to colonise, enter and persist in the host cell through regulation of different protein secretion systems, lipid pathways and other cell surface signal variations leading to infection [7]. Inside the host cell, MAP is known to influence intracellular processes such as apoptosis and regulation of the production of different cytokines. Infected macrophages produce a plethora of cytokines which play multiple roles in the modulation of immune and inflammatory responses. Different cytokines may have multiple actions on different target cells, implying that they may not be specific in function [8]. Cytokines may overlap in action in response to an infection. Early MAP infection is associated with a pro-inflammatory cytokine response involving production of cytokines such as: interferon gamma (IFN-*ƴ*), interleukin 2 (IL-2), IL-6, IL-12 and tumour necrosis factor alpha (TNF-α) whereas the late infection is associated with the production of anti-inflammatory cytokines such as: transforming growth factor - beta (TGF-β), IL-4, IL-10 and IL-13 [9]. MAP interferes with these cellular processes to enable its survival in the intracellular environment and overcome phagocytosis [10].

It has been demonstrated that MAP infection is associated with changes in expression profiles for genes involved in host immune responses that enable its survival and multiplication inside the host macrophages [11]. Mycobacterial genes such as: the non-ribosomal peptide synthatase gene (*pstA*), the conserved polyketide synthase-associated protein (*papA2*), serine/threonine protein kinase G (*pknG*), putative oxidoreductase (*fabG2_2*), probable inositol monophosphatase (*imp*A), possible mycolic acid synthase (*uma*A1), isoprenoid biosynthesis (*gcp*E), catalase-peroxidase (*katG*), probable potassium-transporting ATPase (*kdp*C), among others [12–14]; are believed to play a role in the entry, survival and establishment of MAP in the host macrophages.

Although mice are not natural hosts for MAP, they have been used in previous studies to understand the interactions of the pathogen with the host during early infection [15]. In vitro infection experiments have been performed previously using primary macrophages - monocytes-derived macrophages (MDM) or special cell lines such as RAW 264.7 and J774 among others, to investigate MAP virulence attributes such as viability (survival and multiplication), effect on apoptosis, cytokine production and different gene expression profiles to infer the fate of MAP inside macrophages [16–19]. *Mycobacterium avium* subsp. *paratuberculosis* strains are known to differ in virulence in different host species [3]. Variations in virulence of the different strains could be associated with polymorphisms due to mutations or single nucleotide polymorphisms (SNPs) within the MAP genome. Phylogenetic analysis of the different putative MAP virulence genes is necessary to infer the source of these variations. The differential virulence can be exploited to identify isolates with high immunogenicity that can be used in vaccine development to manage PTB.

Unfortunately, there is a paucity of information concerning virulence of the different MAP strains. The general data situation is particularly poor in many African countries [20]. Reasons for this are mainly the lack of general awareness of the occurrence of this disease in domestic and wild animal populations as well as the infrequent reporting of occurring cases, a consequence of which is the negligence of research on this disease and its aetiology. Finally, it should be noted that PTB is predominantly considered an animal disease and therefore does not have the highest priority in control or research programs as, for example, malaria or tuberculosis. However, this means that further knowledge is lacking, and the pathogen can continue to spread unhindered without being able to assess the consequences for humans and animals.

In this study, questions concerning the behaviour of MAP during infection were addressed. The expression of virulence genes of four field MAP isolates from Uganda during an infection period of 3 to 48 h in RAW 264.7 macrophages as well as the respective cellular cytokine responses to MAP infection were examined. The behaviour of MAP isolates within these time periods was also observed in respect to its replication and viability at the intracellular level. Knowledge of MAP behaviour and virulence mechanisms is important in the design of control and prevention measures for paratuberculosis.

## Materials and methods

### MAP cultivation

**Fig. 1:**
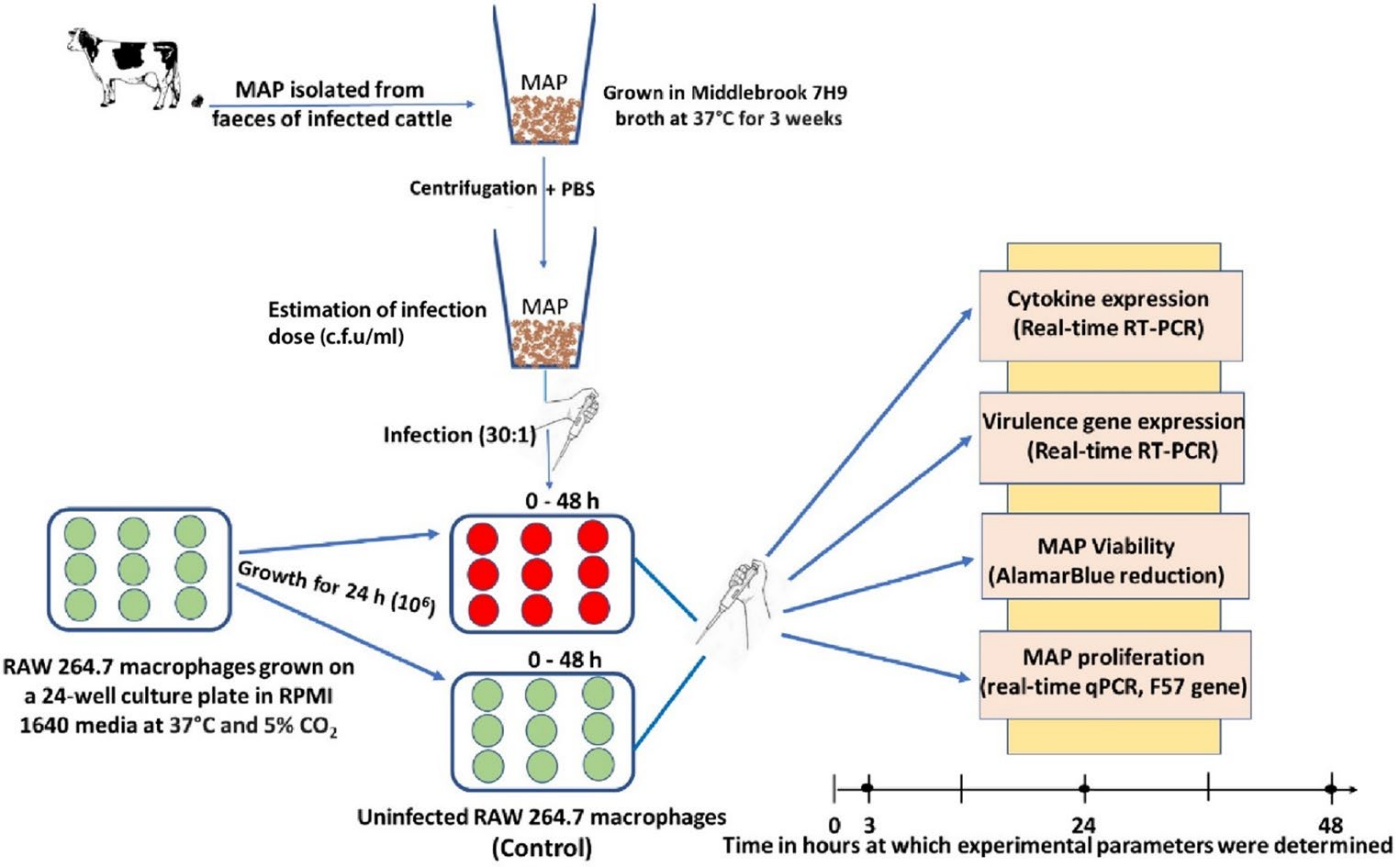
Experimental layout of the 48-h RAW 264.7 murine macrophage in vitro infection model with four different MAP isolates

Samples used in this study were collected in a recent surveillance study of MAP in Uganda [21]. Isolation of MAP was done by culturing faecal samples of MAP positive cattle on Herrold’s Egg Yolk agar slants - HEYM, (Becton, Dickinson and Co., Sparks MD, USA) containing Vancomycin, Amphotericin B and Nalidixic acid with Mycobactin J at 37 °C for a period between 6 weeks to 6 months. All slants that developed colonies typical of MAP were tested to confirm MAP by real-time PCR run on a LightCycler^®^ 96 instrument (Roche Diagnostics, Mannheim, Germany) using primers that target the *IS900* gene (Table 2). Briefly, the PCR mix consisted of 10 µl of LightCycler^®^ 480 Probes Master, 3 µl of PCR water, 0.5 µl of each of the forward and reverse primers (10 µM), 1 µl of the probe (10 µM) and 5 µl of the DNA template. The temperature profile consisted of 10 min of initial denaturation at 95 °C, followed by 45 cycles consisting of denaturation for 15 s at 95 °C, annealing for 30 s at 60 °C and elongation at 72 °C for 35 s as recommended by the manufacturer. MAP positive colonies were sub-cultured on HEYM to achieve pure colonies. A single colony was then inoculated in approximately 5 ml of BBL™ Middlebrook 7H9 broth with glycerol produced by Becton, Dickinson and Co. and supplemented with Mycobactin J (IDvet, Grabels - France) and OADC enrichment (BD BBL™ Middlebrook, Becton, Dickinson and Co.) in a 15 ml centrifuge tube (Sarstedt, Numbrecht, Germany) and incubated at 37 °C for 3 weeks to achieve optimal mycobacterial growth (Heratherm Incubator, ThermoScientific, Langenselbold, Germany) (Fig. 1). Four successfully cultivated MAP isolates: MAP1, MAP2, MAP3 and MAP4; were used in the infection experiments and sequenced for whole genome analysis. These isolates were each obtained from four geographically different districts from within Uganda. The cows were Friesian crosses aged 2 years and above, and were reported to have persistent diarrhoea, with varying body condition scores.

### Sequencing of the four MAP isolates

#### Extraction of MAP DNA

The Cetyltrimethylammonium bromide (CTAB) method was used [22] for the extraction with some modifcations. Briefly, 10 ml of cell suspension was denatured and centrifuged to obtain a pellet. This was homogenized and suspended, mixed and spun serially to obtain the desired mix used for sequencing.

#### Sequence data analysis

To characterise the isolates, MAP DNA was sequenced by whole genome sequencing (WGS) using PacBio^®^ (outsourced) and analysis performed to type the four isolates. Raw reads were assessed for quality using FastQC toolkit [23]. The reads were then processed using fastp; an all-in-one fast processing tool which trims off adapter sequences from reads and also removes bad reads. The trimmed reads were assembled into draft genomes by means of SPADES v3.9.1 using the “-careful” option which performs mismatch correction. The resulting draft assemblies were annotated using PROKKA v1.13.3. To determine the phylogenetic relationship of the four genomes to already known genomes, a phylogenetic tree was constructed including twenty-three complete MAP genomes retrieved from the National Center for Biotechnology Information (NCBI) database. In total, twenty-seven genome sequences were aligned and a phylogenetic tree drawn using MacVector Pro 18.6 software. The parameters used for the phylogenetic tree were: Neighbor Joining for the tree-building method, Best-tree for mode and Kimura 2-parameter for distance.

#### Macrophage preparation

MAP-susceptible RAW 264.7 murine macrophages (CLS Cell Lines Service GmbH, Eppelheim, Germany) were selected for these infection studies. Cultivation was performed under standard conditions of 37 °C and 5% CO_2_ (ESCO CelCulture CO_2_ Incubator; CCL-170 A-8, Singapore) in RPMI 1640 (Sigma- Aldrich, St. Louis, USA) media with the addition of 10% foetal calf serum (FCS, PAA Laboratories, Pasching, Austria), 1% non-essential amino acids, and 1% penicillin/streptomycin (Life Technologies, Grand Island, USA) in 25 cm^2^ and 75 cm^2^ tissue culture flasks (avantor™, Radnor, PA, USA and TPP^®^, Trasadingen, Switzerland). The macrophages were grown for 24 h to achieve confluence before infection.

#### Infection

For the infection, approximately 10 ml of the bacterial cell suspension was centrifuged for 10 min at 3000 g and a single resuspension of the resulting MAP pellet in PBS was performed. Brief homogenization by vortexing was performed to disaggregate the MAP cells. To estimate the number of bacteria in the resulting suspension, its optical density (OD) was measured at 600 nm using a spectrophotometer reader (SPECTROstar Nano S/N 601–1258, BMG LABTECH, Ortenberg, Germany) and dilutions made with PBS while plotting the ODs on a standard calibration curve. A dilution corresponding to an OD of 0.2615 at 600 nm equivalent to 3 × 10^8^ CFU/ml was used for the infection.

Infection experiments were performed in duplicates in 24-well culture plates (Cellstar^®^ Greiner bio-one, Kremsmünster, Austria) with a 24-h RAW 264.7 monolayer of approximately 10^5^−10^6^ cells/ml. Fresh RPMI media was added and incubated with 100 µl each of the previously prepared MAP suspension (3 × 10^8^ CFU/ml) – at a multiplicity of infection (30:1) - and at an atmosphere of 37 °C and 5% CO_2_ (ESCO CelCulture CO_2_ Incubator; CCL-170 A-8, Singapore) for 3, 24, and 48 h. After 3-h incubation, complete removal of the cell culture medium was performed using a pipette followed by washing of the monolayer twice with fresh RPMI 1640 medium to remove extracellular MAP. Subsequently, 1000 µl of fresh RPMI medium was added to each well and incubation was continued at above conditions. For each time interval, one well of non-infected cells was included as a control. A summary of the experimental layout is given in Fig. 1.

#### Viability assay

After the respective time intervals, the cell culture medium was completely disposed and the monolayer was washed with PBS. Subsequently, cell lysis and release of intracellular MAP was performed by adding 0.25% Triton^®^ X-100 (AppliChem, Darmstadt, Germany) at an exposure time of 1 min. The lysates were transferred to 1.5 ml tubes and centrifuged at 5000 g for 2 min. After this step, the resulting pellets were resuspended in 150 µl of fresh Middlebrook 7H9 broth, vortexed and briefly centrifuged. The entire suspensions were next transferred to each well of a 96-well plate done in duplicates.

To determine viability, 15 µl of AlamarBlue (AB) (Life Technologies, Eugene, USA) were added to the suspension per well (ratio of 10:1). Darkened incubation for 24 h (optimal time to reduce the oxidized AB) was followed by absorbance measurements at wavelengths of 570 and 600 nm using spectrophotometer reader (SPECTROstar Nano, S/n 601–1258 – BMG LABTECH, Ortenberg, Germany). The calculation of the percentage AlamarBlue reduction was performed using a suitable formula [24]. To determine the AlamarBlue reduction at 570 nm (AR_570_), the correction factor (Ro) was first calculated as a ratio of the difference between the mean absorbance of media and media plus AlamarBlue at 570 nm to its absorbance at 600 nm, which was then applied to the formula as shown in Table 1.

**Table 1:** Calculation for correction factor (R_o_)

	Ab 570 nm	Ab 600 nm (Reference)
150 µl of media + 15 µl AlamarBlue	0.340	0.468
150 µl media	0.056	0.059
Difference	0.284	0.409

$$Correction\;factor\;\left(R_0\right)=Absorbance\;at\;570\;nm/Absorbance\;at\;600nm$$

$$0.284/0.409=0.694$$

Formula for percentage reduction of AB.

$$AR_{570}=A_{570}-\left(A_{600}\times R_o\right)\times100$$

Where;

- AR_570_ – AlamarBlue reduction at 570 nm.
- A_570_ - Absorbance at 570 nm.
- A_600_ - Absorbance at 600 nm.
- R_o_ - Correction factor.

#### Expression of cytokines and virulence genes during infection

At the end of the respective time interval, the medium was completely aspirated from the respective wells and the monolayer was washed with PBS. This was followed by the addition of lysis buffer containing 2-mercaptoethanol (Sigma Aldrich) and manual homogenization by pipetting to release intracellular MAP. Ribonucleic acid (RNA) from 300 µl of cell lysate were extracted using the PureLink™ RNA Mini Kit (Invitrogen, Carlsbad, CA, USA) according to the manufacturer’s recommendations.

This was followed by the performance of real-time RT-PCR for cytokine and virulence gene expression using a SYBR Green RT-PCR kit (Sigma Aldrich) on a 96-well, 0.2 ml, QuantStudio™ 5 PCR Cycler (ThermoFischer Scientific, Waltham, USA). The reaction total volume was 25 µl, containing 1 µl of each primer (10 µM), 0.025 µl Reference Dye, 0.125 µl reverse transcriptase (M-MLV RT), 12.5 µl of SYBR Green Taq Ready-Mix and 3 µl of the template DNA. The temperature profile was a 2-min initial activation at 94 °C, followed by 40 cycles of denaturation for 15 s at 94 °C, annealing/extension for 1 min at 60 °C as recommended by the manufacturer. Reference (housekeeping) genes *Ig2* and cancer susceptibility candidate 3 A/metastatic lymph node 51 (casc3A/mln51a) for MAP virulence genes and RAW 264.7 cytokine normalization respectively were also run. Primers for the specific cytokines, anti-apoptotic, MAP identification, quantification and virulence genes are given in Table 2.

**Table 2:** Primers used in this study

Gene name	Primer sequence (5’– 3’) Forward	Primer sequence (5’– 3’) Reverse	Size (bp)	Source
**MAP Identification**				
IS900	TACCGCGGCGGAGGCAAGAC	CGGAACGTCGGCTGGTCAGG	139	CP053068.1
Probe	FAM- CCTGATCGGCGATGATCGCAGCGTCTT - BHQ			
**MAP enumeration**				
*F57*	GCCCATTTCATCGATACCC	GTACCGAATGTTGTTGTCAC	254	Accession no. X70277
*Probe*	6FAM- CAATTCTCAGCTGCAACTCGAACACAC - BHQ1			
**Cytokines**				
*IL-12P40*	TCAGACCAGAGCAGTGAGGT	GCAGGTGAAGTGTCCAGAAT	243	[25]
*IFN-ƴ*	GGTCATTCAAAGGAGCATGGA	CTGCCATTCAAGAACTTCTGA	55	
*TNF-α*	CGCATTGCAGTCTCCTACCA	GGGCTCTTGATGGCAGACA	238	
*TGF-β*	TGAGCCAGAGGCGGACTACT	TGCCGTATTCCACCATTAGCA	169	
*IL-10*	CTTGTCGGAAATGATCCAGTTTT	TCAGGCCCGTGGTTCTCA	305	
*IL-6*	GCTGCTCCTGGTGATGACTTC	GGGTGGTGTCATTTTTGAAATCTT	236	
*BCL2-1*	CACCCAGGGACAGCATA	CGTCCCGGAAGAGTTCATTC		[26]
**Virulence genes**				
*impA*	CTGACCTGGTTGCCGTTC	GCGGGATTTCGTTCTTGC	76	[27]
*katG*	CAACCAGGGCAAGTTCGTC	AAGCGGTCGTTGTTCATCAC	66	
*kdpC*	CACCGTTCGTGAGCCTCT	ATCTGGCCGAGCGAATAGT	76	
*papA2*	GGCGTTCCCACAGAATCC	CAGACACATCGCCCTGAC	73	
*umaA1*	TTGACCTACACCCAGAAGCAG	AACCGTAAATCGCTCATCG	66	
***Housekeeping genes***				
*1g2*	AAACGATTTGAACAAGGTGCTC	CGAATAGGGCGCTGAATG	119	[27]
Casc3A/mln-51a	CTGCCTTGTCTTTTGCAGTA	TGCAAGTACAGGAGCAGAAT		[28]

#### Intracellular MAP quantification

At the end of the respective time interval, the medium was completely aspirated from the respective wells and the monolayer was washed with PBS. This was followed by the addition of Buffer AL and Proteinase (Qiagen - Hilden, Germany) for cell lysis and manual homogenization by pipetting to release intracellular MAP. Deoxyribonucleic acid (DNA) from 560 µl of cell lysate was extracted using the QIAamp^®^ DNA Blood Mini Kit 250 (Qiagen) according to the manufacturer’s recommendations. This was followed by quantification by performing real-time qPCR with LightCycler^®^ 480 Probes Master (Roche Diagnostics, Mannheim, Germany) on a QuantStudio™ 5 PCR Cycler (ThermoFischer Scientific). The reaction total volume was 20 µl, containing 1.0 µl of each primer (10 µM), 0.5 µl (10 µM) of the probe, 10 µl of Light Cycler 480 Probe Master and 5.0 µl of template/MAP DNA standard. The temperature profile was: initial denaturation at 95 °C for 5 min, followed by 45 cycles of denaturation at 95 °C for 10 s, annealing and extension at 60 °C for 40 s as recommended by the manufacturer, using primers that target the F57 gene (Table 2). For this, a synthetic molecular MAP F57 DNA standard of known copy numbers of the F57 gene produced by Invitrogen (Carlsbad, CA, USA) was used and a standard curve was drawn using serial dilutions (10^7^ − 10^1^) to calculate the number of MAP copies.

#### Data analysis

Data for relative gene expression for both cytokines and virulence gene as well as viability was entered into MS Excel and analysed. Further analysis was done to determine whether there were significant differences among the cytokine and gene expression profiles of the four MAP isolates at different time points *(p-value < 0.05*) by one-way analysis of variance (ANOVA) using the R statistical software [29]. Relative gene expression (∆∆C_t_) was calculated as previously reported by Taylor and others [28]:

$$\begin{array}{c}\triangle\triangle C_t={\left(C_{t\;gene\;of\;interest\;}-C_{t\;reference\;gene}\right)}_{treated\;sample}\\-{\left(C_{t\;gene\;of\;interest}-C_{t\;reference\;gene}\right)}_{untreated\;sample}\end{array}$$

Twice up- or down-regulated genes were considered significant [30].

## Results

### Virulence genes and cytokines expression during infection

**Fig. 2:**
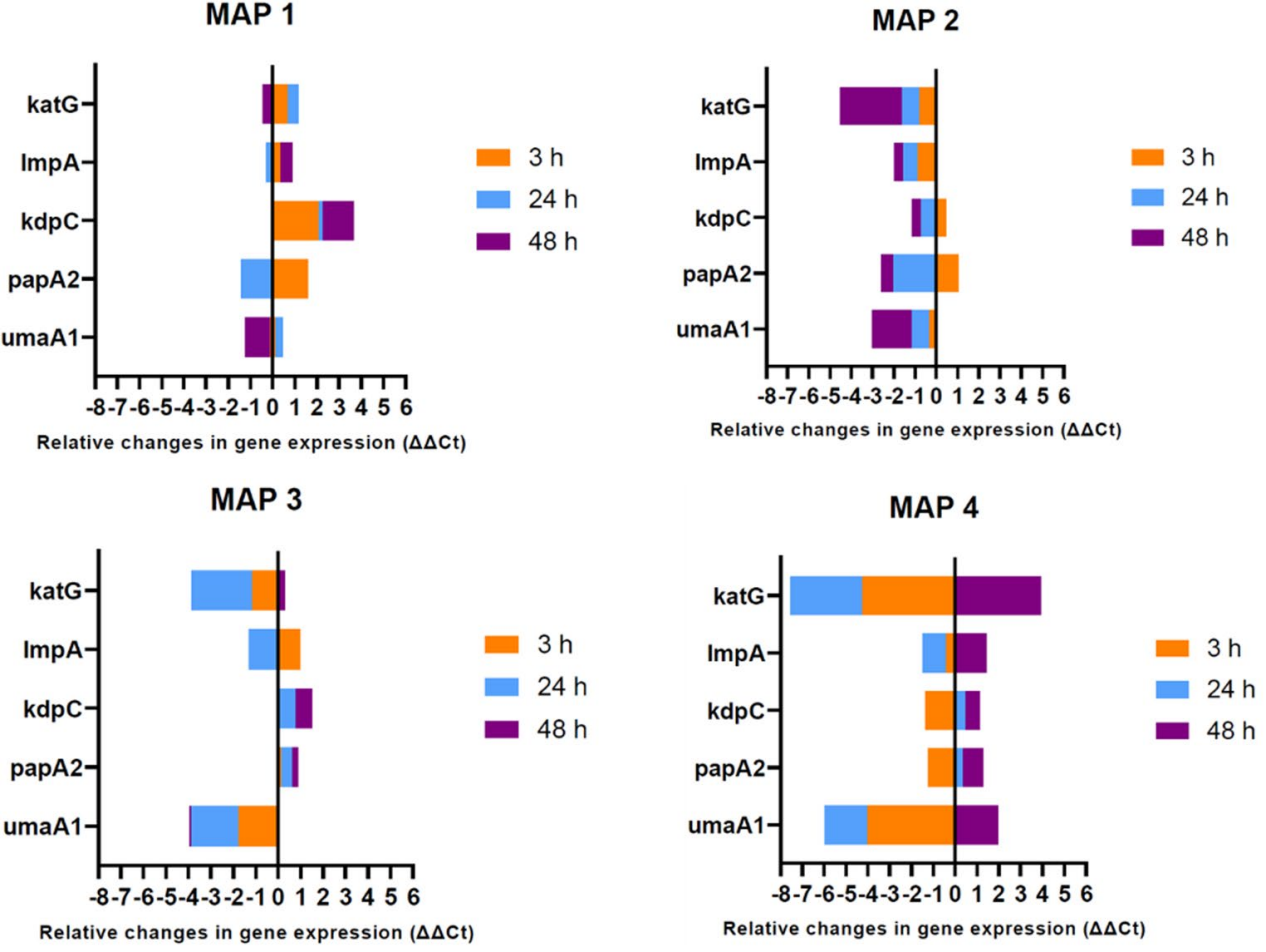
Relative changes in virulence gene expression profiles of MAP isolates after 3 h, 24 h and 48 h of infection

The four MAP isolates exhibited notable variations in the relative expression of the various virulence genes within 3 h, 24 h, and 48 h pi (Fig. 2). Statistical analysis of the virulence gene expression profiles however revealed no significant differences among the isolates at the different time points (*ANOVA*,* p-value > 0.05*), though MAP4 had the highest mean values for the virulence gene expression across the treatments. In general, MAP1 showed an initial up-regulation of most of the virulence genes, followed by down-regulation of *papA2* and *umaA1* genes and a final up-regulation of the *kdpC* gene. MAP2 showed a down-regulation of all genes after 24 h pi. MAP3 on the other hand, showed pronounced down-regulation of the *katG* and *umaA1* genes at all time points post-infection (pi). The rest of the genes showed variable levels in their relative expression at different time points. Likewise, MAP4 exhibited pronounced down-regulation of *katG* and *umaA1* genes up to 24 h pi. The other genes showed variable degrees of relative expression for the different time points (3 h, 24 h and 48 h) pi; all the genes were up-regulated after 24 h pi.

**Fig. 3:**
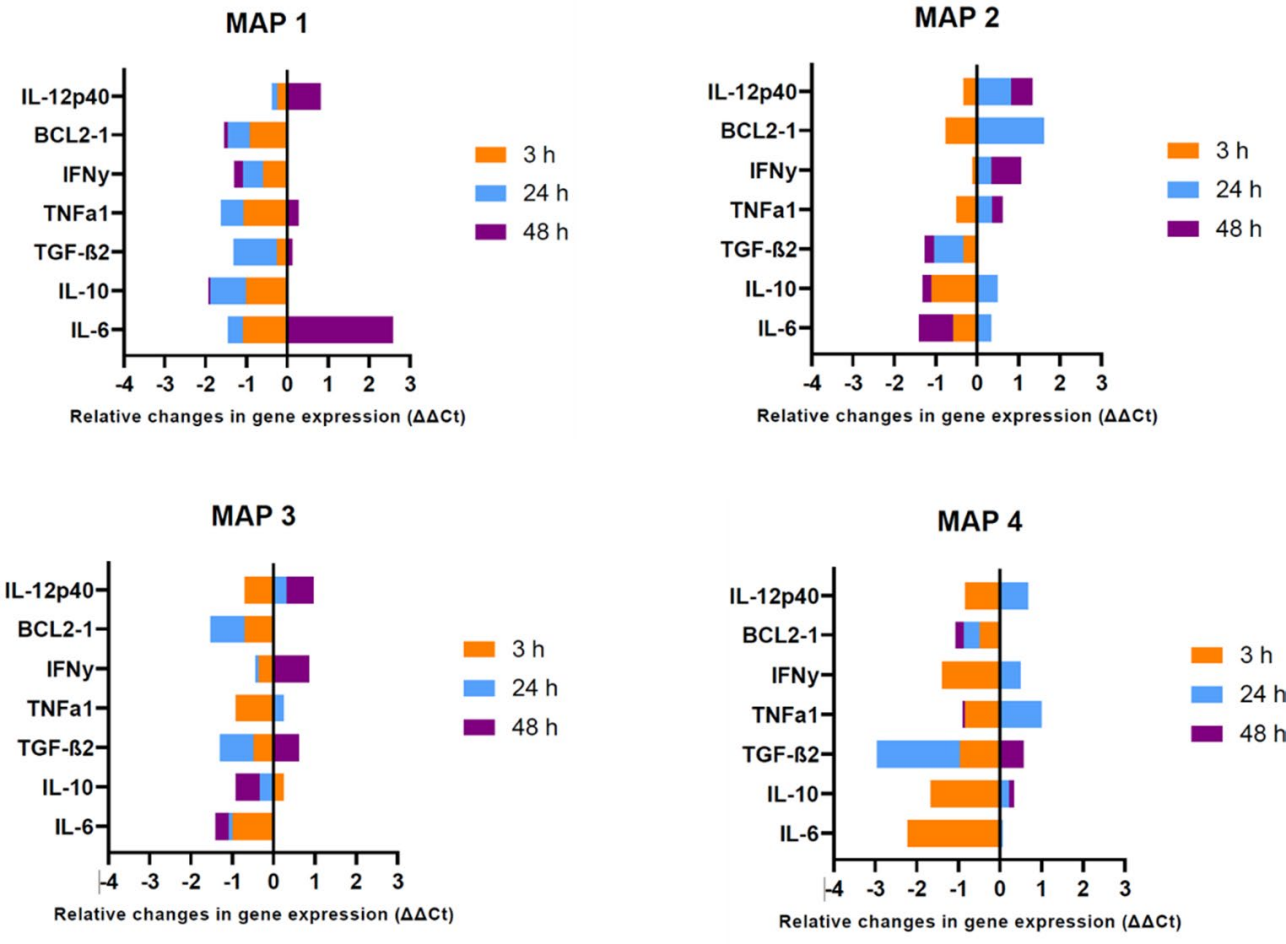
Relative changes in cytokine expression profiles of MAP isolates at 3 h, 24 h and 48 h post infection

For cytokine expression, the very non-homogeneous picture regarding the up- and down-regulation of the individual cytokines in macrophages infected with the different MAP isolates was striking (Fig. 3). During the 3-h infection period, high mean values for cytokine expression were observed in response to MAP3, with statistically significant differences observed across all the isolates (*p* < 0.01). During the 24-h interval, macrophages infected with MAP2 exhibited the highest mean values of cytokine expression while those infected with MAP4 had the least mean expression values. *Post hoc* tests (Turkey’s hsd) indicated significant differences in cytokine expression in macrophages infected with MAP2 and MAP4, (*p* = 0.025 though no significant differences were exhibited at 48 h p.i (*p* = 0.231).

For macrophages infected with MAP1, all cytokines were down-regulated between 3 h and 24 h pi though there was a remarkable up-regulation of IL-6 at 48 h pi. Macrophages infected with MAP2 showed relative up-regulation of the pro-inflammatory cytokines (IFN-*ƴ*, TNF-α, IL-12p40) and a down-regulation of the anti-inflammatory cytokines (IL-10, TGF-β) at 24 h pi. Interleukin – 6, which is known to have pro-inflammatory properties, was also down-regulated at this time point. Macrophages infected with MAP3 exhibited a degree of variability in the relative cytokine expression with no clear-cut differences between the 3 h and 24 h pi; though at 24 h pi, IL-12p40, IFN-*ƴ* and TGF-β were up-regulated. Macrophages infected with MAP4 on the other hand showed a clear down-regulation for all cytokines at 3 h pi. TGF-β was further down-regulated at 24 h pi but was later up-regulated at 48 h pi. The anti-apoptotic (*BCL-2*) gene was down-regulated in macrophages infected with MAP1, MAP3 and MAP4; it was only up-regulated in MAP2 at 24 h pi.

### Quantification of intracellular MAP

**Fig. 4:**
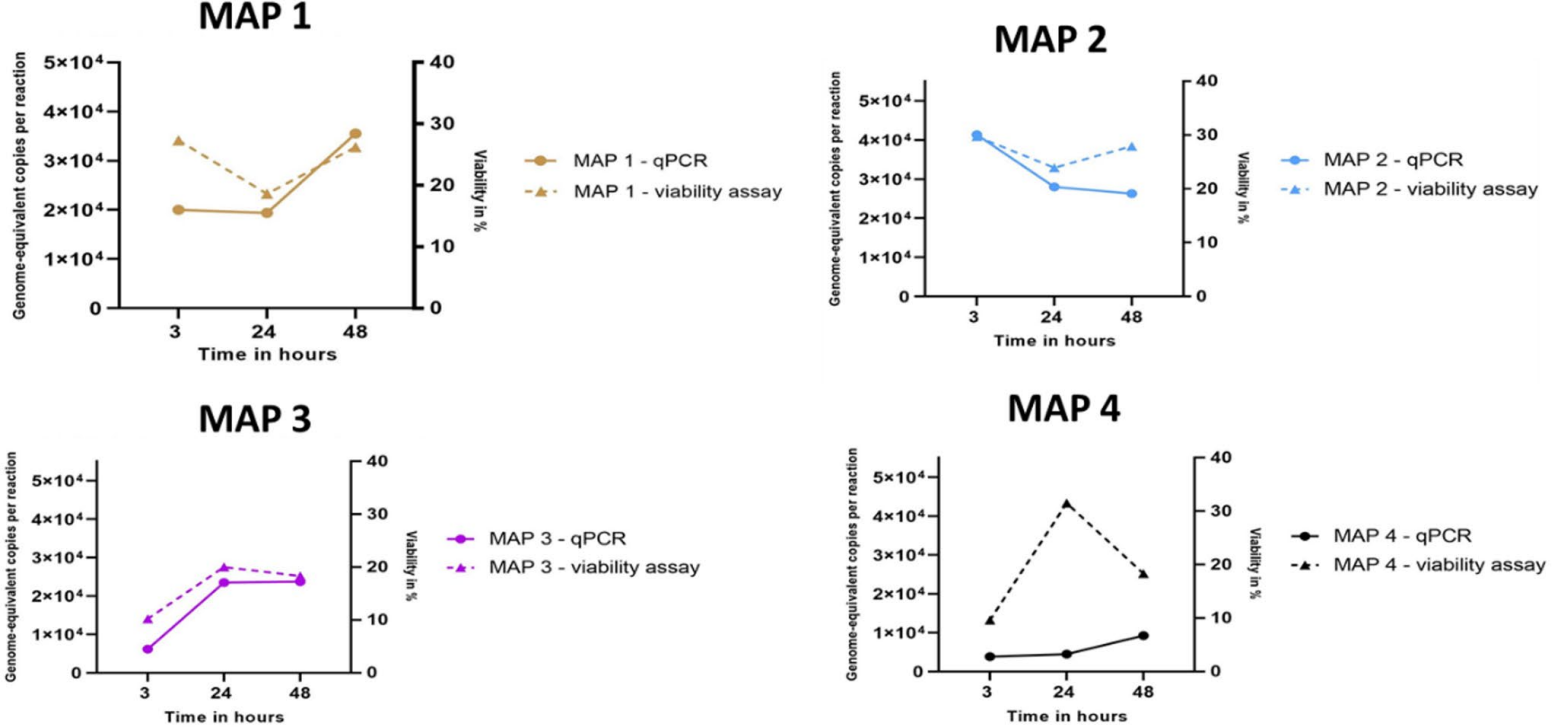
Intracellular MAP quantities and viability in macrophages at different time points post infection

All MAP isolates exhibited replication evidenced by increase in intracellular DNA albeit at a low rate within 48 h (less than 0.5 log_10_ levels). Only MAP2 showed a slight reduction in the amount of the intracellular DNA after 48 h pi (less than 0.5 log_10_ levels). In general, the change in intracellular MAP DNA at all time points and in all isolates was considered not appreciable (Fig. 4).

### Viability

The viability study revealed that the percentage reduction of AlamarBlue and thus cell activity followed that of MAP DNA quantities, and this was true for MAP1, MAP2 and MAP3 for the 48-h infection period. MAP4, on the other hand, showed a reduction in MAP viability against a slight increase in MAP quantity at 24 h pi. In addition, MAP4 had low amounts of MAP intracellularly compared to the other isolates (Table 3). Statistical analysis however revealed no significant differences in viability among the isolates at the different time points *(p-value > 0.05).*

**Table 3:** Mean values for MAP viability in RAW 264.7 macrophages at different time points post infection

Isolate	% reduction of AlamarBlue pi (MAP viability, *p* > 0.05)		
	3 h	24 h	48 h
MAP1	27.3	18.6	26.2
MAP2	29.7	23.9	27.9
MAP3	10.2	20	18.3
MAP4	9.6	31.5	18.3

### MAP genome sequence alignment and phylogenetic tree

**Fig. 5:**
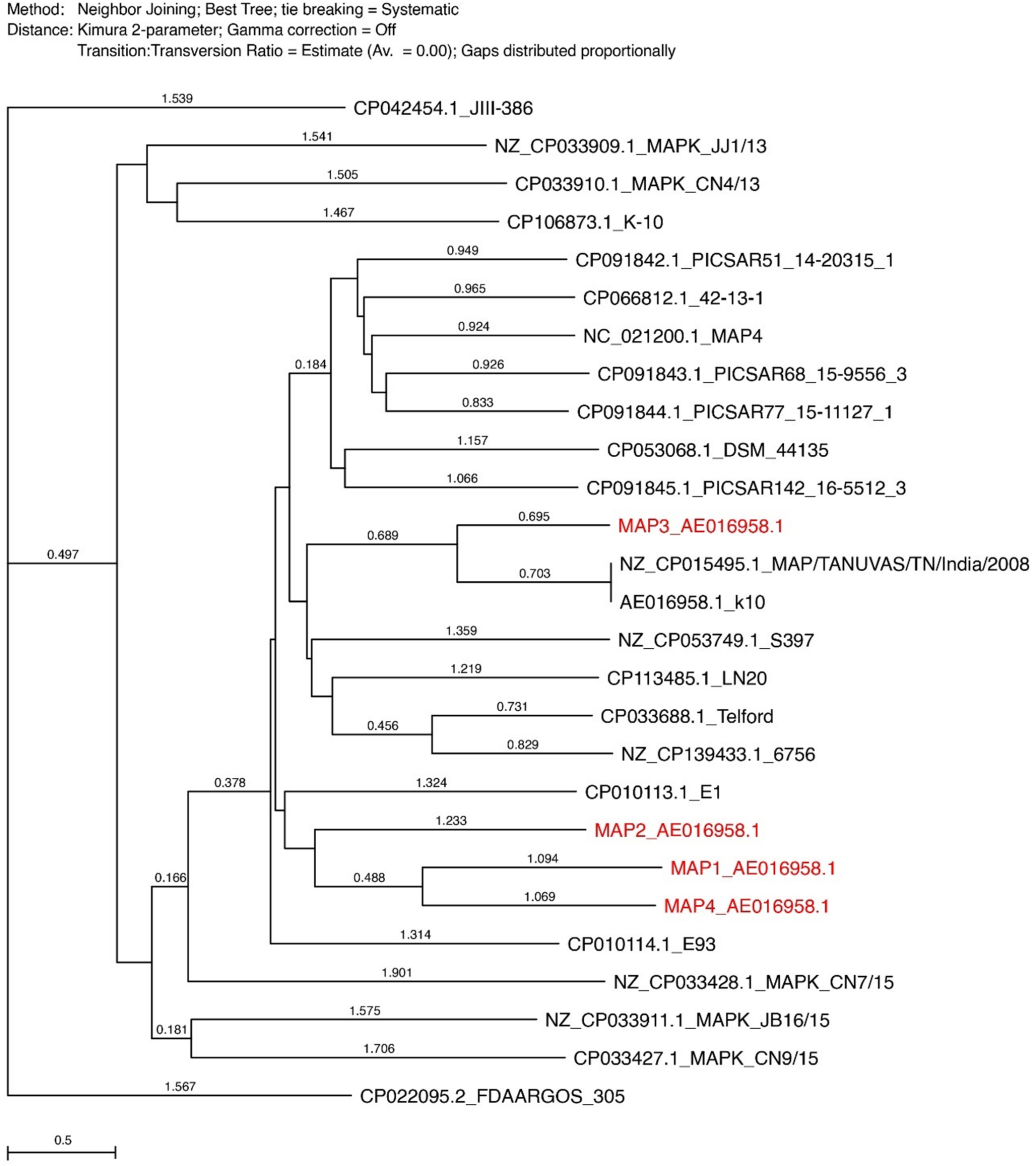
Phylogenetic tree of the four study MAP genomes (MAP1, MAP2, MAP3 and MAP4) in comparison with other mycobacterial strains and the reference MAP_K10 cattle strain (AE016958.1) based on whole genome sequences

The phylogenetic tree drawn from the four study genomes and other genomes with reference to the cattle MAP_K10 complete genome revealed distinctive groupings (Fig. 5). Three of the isolates of this study (MAP1, MAP2 and MAP4) and another cattle isolate from Egypt (CP010113.1), clustered in one clade. Isolate MAP3 on the other hand, grouped differently from the three study genomes but clustered with the reference MAP_K10 (AE016958.1) and another cattle isolate of Indian origin (NZ_CP015495.1).

## Discussion

To bring paratuberculosis (PTB) out of its neglected position, intensive and targeted research is needed to create awareness of its danger and to develop and establish targeted diagnostic and control strategies. Understanding the behaviour of MAP inside the host cells and how it causes subsequent infection would be helpful for effective diagnosis and control of PTB. In this study, we observed the pattern of regulation of some putative virulence genes of MAP, anti-apoptotic gene (*BCL-2*), MAP survival inside macrophages as well as the behaviour of some cytokines involved in inflammatory processes in RAW 264.7 macrophages infected with different MAP isolates. Four cattle MAP isolates were used in this study: MAP1, MAP2, MAP3 and MAP4.

Different MAP strains have been shown to differ in their ability to survive in monocytes-derived macrophages (MDM). In one of the earlier studies, it was observed that MAP strains of distinct genotype varied in their survival in MDM [26]. These MAP strains belonged to different hosts, and it was concluded that MAP strains express differential virulence due to host associations. In another study, MAP strains of bovine and human origin were used to infect MDM. An anti-inflammatory response was observed with the up-regulation of IL-10 and down-regulation of the TNF-α [31]. Again, in another in vitro infection model, an up-regulation of cytokines including IL-6 and IL-10, which favours MAP survival intracellularly was exhibited [32]. Observations were also made in a study involving MAP infection with RAW 264.7 murine macrophages, in which it was demonstrated that MAP suppresses a pro-inflammatory response during infection [33]. Macrophages infected with MAP are known to produce different cytokines in response to the infection. Cytokines are known to play diverse roles in response to an infection and may overlap in function.

In the current experiment, macrophages infected with MAP1 isolate exhibited significant up-regulation of IL-6 at 48 h pi. Interleukin-6 is known to have pro-inflammatory properties, however, in MAP infection the anti-inflammatory attribute is predominant [34]. The behaviour of MAP1 regarding IL-6 is suggestive of the MAP survival strategy inside macrophages as was also observed in an earlier study [31]. Other cytokines including the tumour necrosis factor-alpha (TNF-α), interferon-gamma (IFN-*ƴ*) and interleukin-12p40 among the pro-inflammatory cytokines; IL-10 and transforming growth factor-beta (TGF-β) among the anti-inflammatory cytokines [35, 36] varied considerably in regulation (ANOVA, *p-value < 0.05*). Conversely, macrophages infected with MAP2 displayed a characteristic response pattern featuring up-regulation of pro-inflammatory cytokines and down-regulation of the anti-inflammatory cytokines. For macrophages infected with MAP3 and MAP4, their cytokine expression profile was not suggestive of a particular trend, however; they both showed an initial down-regulation of all cytokines and eventual up-regulation later in the infection. The anti-apoptotic gene (*BCL-2*) was generally down-regulated in response to all isolates except for macrophages infected with MAP2, where it was up-regulation at 24 h pi. MAP is known to interfere with normal macrophage function by inhibiting apoptosis, thereby promoting its survival in macrophages [37]. Isolate MAP2 demonstrated more ability to survive inside macrophages compared to the other isolates in this regard. The other MAP isolates except MAP2 favoured a pro-apoptotic profile contrary to the expected behaviour of MAP. A similar trend was observed in an earlier study involving MAP infection in murine macrophage model [38]; and this could point to the variations in virulence. However more studies are required to investigate other host/pathogen factors that interact to determine infection outcomes.

Some of the probable MAP virulence genes investigated in this study included: *kdpC*, *katG*, *papA2*, *impA* and *umaA1*. These genes are involved in cellular pathways that play different roles in enabling MAP to evade the host defence mechanisms and cause infection in a susceptible host [12]. In the current experiment with RAW 264.7 macrophages, although no statistically significant differences were exhibited generally for all isolates (*P* > 0.05), there were remarkable differences in the relative expression of the individual virulence genes for all the four isolates post infection. In particular MAP2 showed significant down-regulation of all virulence genes after the 24 h pi contrary to its cytokine expression profile that exhibited survival ability. This implies that there could be other virulence factors mediating the virulence behaviour earlier observed in this isolate. On the other hand, MAP4 exhibited marked down-regulation of all virulence genes in the initial 24 h, but later they were all up-regulated particularly *katG* and *umaA1* genes. A similar trend was observed in MAP3 though to a less extent. The expression of the different virulence genes is an attempt by MAP cells to survive in the macrophage environment. The *umaA1* gene was reported to play a role in organ colonisation and cell biosynthesis [39]. The increased expression of the *katG* gene, a probable catalase/peroxidase, is believed to be a response to the oxidative stress encountered by MAP within the macrophage environment to protect the bacterial cells against hydrogen peroxide as was reported in an earlier work [14]. In contrast, MAP1 did not show marked differences in gene expression between the up- and down-regulation profiles though the *kdpC* gene was up-regulated throughout the experimental period. The *kdpC* gene, a probable potassium-transporting ATPase C chain has been shown to be associated with organ colonisation and granuloma formation in MAP [12]. These observations are suggestive of possible differences in virulence mechanisms of these strains.

MAP cell viability and quantities inside macrophages varied considerably among the isolates though with an overall increase at 24 h pi, as an indication of successful replication inside macrophages. MAP1 in particular exhibited significant increase in number of MAP cells intracellularly after 24 h of infection. MAP4 on the other hand, exhibited low levels of survival inside the macrophages at all time points and the viability dropped at 24 h pi unlike the other isolates which had significant amounts of MAP surviving inside macrophages up to 48 h pi. All MAP isolates except MAP4 had significantly high numbers of MAP copies (over 2 × 10^4^ genome equivalents) at 24 h pi; this was an indication of variable survival abilities of the different isolates. Generally, there was minimal increase in the amount of MAP cells inside macrophages for all strains at all time points implying a low multiplication rate. This is due to MAP having a long generation time of over 20 h [40]. Hence, further observations could not be made beyond the 2-day incubation period. We could not afford to go beyond the 48 h of infection, as the RAW 264.7 macrophages were overgrown after this time point and were no longer a confluent monolayer. For future studies, we recommend use of macrophages with a slow replication rate to avoid this problem and allow for longer experimental period if desired. It is also proposed that models that mimic the in vivo host environment be used for a better understanding of how MAP organisms interact within the natural intracellular environment.

From the phylogenetic analysis, MAP3 clustered with the Indian cattle isolate (CP015495.1) and the reference MAP_K10 strain; the former (CP015495.1) was previously used to generate recombinant antigens for MAP diagnostics [41]. This highlights the potential use of the present isolates in the production of antigenic and immunogenic responses which can be exploited for MAP diagnostics and vaccine development. On the other hand, study isolates (MAP1, MAP2 and MAP4) grouped with an isolate from Egypt (CP010113.1). These isolates, though obtained from different geographical locations, exhibited evolutionary relatedness. The Ugandan isolates also have evolutionary relationships with the two isolates from India and Egypt as well as with the reference MAP_K10 strain. Further studies need to focus on these isolates to determine their possible use in the development of PTB diagnostic and management tools.

## Conclusion

The four MAP isolates demonstrated varying degrees of virulence regarding cytokine, apoptotic and virulence gene responses. However, MAP1, MAP2 and MAP3 exhibited higher ability to survive and replicate inside macrophages compared to MAP4 isolate. These assays present a way of assessing the behaviour of MAP inside macrophages. The use of cytokines and virulence gene expressions to study MAP behaviour inside the host macrophages is an appropriate attempt to decipher MAP pathogenesis. Since MAP is a fastidious organism and paratuberculosis takes a long time to develop into clinical disease, these in vitro models will aid the understanding of MAP pathogenesis.

## Supplementary Information

Below is the link to the electronic supplementary material.

Supplementary Material 1 (XLSX 30.6 KB)

## Data Availability

All data are available in the manuscript text and in the supplementary information files. Whole Genome Sequence data for MAP isolates is available on request from the corresponding author at 10.5281/zenodo.15935180.
